# Pharmacologically Inhibiting Glycogen Synthase Kinase-3β Ameliorates Renal Inflammation and Nephrotoxicity in an Animal Model of Cisplatin-Induced Acute Kidney Injury

**DOI:** 10.3390/biomedicines9080887

**Published:** 2021-07-25

**Authors:** Chung-Hsi Hsing, Cheng-Chieh Tsai, Chia-Ling Chen, Yu-Hui Lin, Po-Chun Tseng, Rahmat Dani Satria, Chiou-Feng Lin

**Affiliations:** 1 Department of Anesthesiology, Chi-Mei Medical Center, Tainan 710, Taiwan; hsing@mail.chimei.org.tw; 2 Department of Medical Research, Chi-Mei Medical Center, Tainan 710, Taiwan; 3 Department of Anesthesiology, College of Medicine, Taipei Medical University, Taipei 110, Taiwan; 4 Department of Nursing, Chung Hwa University of Medical Technology, Tainan 703, Taiwan; cctsai@mail.hwai.edu.tw; 5 Department of Long Term Care Management, Chung Hwa University of Medical Technology, Tainan 703, Taiwan; 6 School of Respiratory Therapy, College of Medicine, Taipei Medical University, Taipei 110, Taiwan; chialing66@tmu.edu.tw; 7 Graduate Institute of Clinical Medicine, College of Medicine, National Cheng Kung University, Tainan 701, Taiwan; cflin2016@yahoo.com; 8 Department of Microbiology and Immunology, School of Medicine, College of Medicine, Taipei Medical University, Taipei 110, Taiwan; pctseng@tmu.edu.tw (P.-C.T.); dr.dani.satria@gmail.com (R.D.S.); 9 Core Laboratory of Immune Monitoring, Office of Research & Development, Taipei Medical University, Taipei 110, Taiwan; 10 International Ph.D. Program in Medicine, College of Medicine, Taipei Medical University, Taipei 110, Taiwan; 11 Department of Clinical Pathology and Laboratory Medicine, Faculty of Medicine, Public Health and Nursing, Universitas Gadjah Mada, Yogyakarta 55281, Indonesia; 12 Clinical Laboratory Installation, Dr. Sardjito Central General Hospital, Yogyakarta 55281, Indonesia; 13 Graduate Institute of Medical Sciences, College of Medicine, Taipei Medical University, Taipei 110, Taiwan

**Keywords:** cisplatin, AKI, GSK-3β, renal inflammation, nephrotoxicity

## Abstract

The adverse effect of cisplatin administration causes acute kidney injury (AKI) following renal inflammation and nephrotoxicity, characterized by proximal tubular cell apoptosis and necrosis. Pro-apoptotic and pro-inflammatory roles of glycogen synthase kinase (GSK)-3β have been reported. This study investigated the therapeutic blockade of GSK-3β in cisplatin-induced AKI. A renal cisplatin nephrotoxicity model showed activation of GSK-3β in vivo, particularly in proximal tubular epithelial cells. Pharmacologically inhibiting GSK-3β abolished cisplatin nephrotoxicity, including proximal tubular injury, cell cytotoxicity, and biochemical dysfunction. Additionally, GSK-3β inhibitor treatment ameliorated renal inflammation by reducing immune cell infiltration, cell adhesion molecule expression, and pro-inflammatory cytokine/chemokine production. Cisplatin treatment caused GSK-3β activation in vitro in the human renal proximal tubular epithelial cell line HK-2, whereas either pharmacological administration of GSK-3β inhibitors or genetic transduction of GSK-3β short-hairpin RNA impeded cisplatin-induced cytotoxicity. These results indicate that cisplatin activates GSK-3β followed by GSK-3β-mediated renal inflammation and nephrotoxicity, contributing to AKI.

## 1. Introduction

Cisplatin, a highly effective chemotherapeutic drug, is widely used to treat various cancers, including sarcomas, lymphomas, small-cell lung cancer, head and neck cancer, ovarian cancer, and testicular cancer [1]. Following treatment, cisplatin accumulates in cells and can cross-link with DNA to disrupt the double-helical structure of DNA [2]. Then, cisplatin can induce mitochondrial damage, cell cycle arrest, and cell death [3]. Regarding its cytotoxicity to normal tissues, nephrotoxicity is a major side effect that limits cisplatin treatment. Cisplatin induces renal tubular cell death, which leads to nephrotoxicity [3,4,5]. Approximately one-third of patients treated with cisplatin develop acute kidney injury (AKI). The strategy is to synthesize safe analog carboplatin to avoid adverse effects. However, the plasma half-life of carboplatin is longer than that of cisplatin, and carboplatin exerts unexpected side effects [6]. Another approach for minimizing cisplatin-induced AKI is intensive hydration with phosphate-buffered saline [7]. Unfortunately, despite hydration, cisplatin-induced AKI still occurs.

The mechanisms that underlie cisplatin-induced nephrotoxicity are complicated. Current studies have demonstrated that the exposure of tubular cells to cisplatin activates multiple signaling pathways, including DNA damage and ATR/ATM/p53 activation [8]; reactive oxygen species (ROS) generation [9]; epidermal growth factor receptor (EGFR) signaling [10], including Src, protein kinase C (PKC)-δ, and mitogen-activated protein kinase (MAPK) activation. Together, these pathways lead to tubular cell death [5,11]. In addition to the cytotoxic effects directly caused by cisplatin, an inflammatory response indirectly occurs during cisplatin-induced AKI that may exacerbate renal tissue damage and cause inflammation-associated nephrotoxicity [12]. Inflammatory processes, including the production of cytokines tumor necrosis factor (TNF)-α, interleukin (IL)-6, and interferon (IFN)-γ, inflammatory mediator generation, and immune cell infiltration, facilitate cisplatin-induced AKI [13]. Additionally, cisplatin induces renal vascular cell injury and results in decreased blood flow and ischemic injury of the kidneys, which decreases the glomerular filtration rate [5]. Together, tubular cell death, inflammation, and ischemia result in the loss of renal function during cisplatin-induced AKI. However, how to simultaneously kill tumors without cytotoxic effects on kidneys remains a complex puzzle of cisplatin action.

Glycogen synthase kinase (GSK)-3β, a serine/threonine kinase, facilitates apoptotic signaling and cellular inflammatory activation in response to various stimuli, such as growth stress, pathogens, anticancer agents, and cytokines [14]. In renal ischemic injury, active GSK-3β may be pro-apoptotic by causing the activation of the caspase-3 cascade through Bax to promote renal cell apoptosis [15]. Furthermore, in addition to cytotoxicity, GSK-3β facilitates inflammatory activation in response to the stimulation of TLRs and cytokines, such as TNF-α, IL-6, and IFN-γ [16,17,18]. As summarized in a review of renal injury pathogenesis [19], the pro-inflammatory and pro-apoptotic roles of GSK-3β are speculated to be pathogenic and can be therapeutically targeted. Certainly, pharmacologically inhibiting GSK-3β confer the possible therapeutic effects against renal injury in vivo in the rat model of ischemia [15] and the murine models of endotoxemia [20] and pharmacological stimulation, including nonsteroidal anti-inflammatory drug diclofenac and mercuric chloride [21,22]. Regarding cisplatin-induced AKI usually causes renal tubular cell death and renal inflammation [5,19], this study investigated the therapeutic effects on cisplatin-induced AKI by pharmacologically inhibiting GSK-3β activation.

## 2. Materials and Methods

### 2.1. Antibodies and Reagents

Antibodies against phospho-glycogen synthase (GS) (Ser641) (Cat.# 3891), GS (Cat.# 3886), GSK-3β (Cat.# 9832), and GAPDH (Cat.# 2118) were purchased from Cell Signaling Technology (Beverly, MA, USA). Anti-phospho-GSK-3β (Tyr216) (Cat.# NB10081946) antibody was purchased from Novus Biologicals (Littleton, CO, USA). Alexa Fluor 488-labeled anti-mouse CD3 (Cat.# 100210), Gr-1 (Cat.# 108417), CD11b (Cat.# 101217), and CD54 (Cat.# 116112) were obtained from BioLegend (San Diego, CA, USA). Horseradish peroxidase (HRP)-conjugated goat anti-mouse (Cat.# 62-6520), goat anti-rabbit (Cat.# 65-6120), goat anti-rat (Cat.# 31470), and donkey anti-goat (Cat.# A15999) IgG antibodies were obtained from Invitrogen (Carlsbad, CA, USA). GSK-3 inhibitors 6-bromo-indirubin-3′-oxime (BIO, Cat.# B1686), SB216763 (Cat.# S3442), and SB415286 (Cat.# S3567) were purchased from Sigma-Aldrich (St. Louis, MO, USA). All drugs were assessed for their cytotoxic effects in cells using cytotoxicity assays before the experiments were performed. Non-cytotoxic dosages were used in this study. By utilizing the online tools, adapted from the Research Collaboratory for Structural Bioinformatics Protein Data Bank (RCSB PDB) 1UV5 (https://www.rcsb.org/3d-view/1UV5, accessed on 20 July 2021) (Piscataway, NJ, USA,), the secondary structure of the GSK-3β:BIO complex was modified and drawn according to the previous works [23,24]. Furthermore, the wire-frame structure of BIO was adapted and modified from PubChem SID 833555 (https://pubchem.ncbi.nlm.nih.gov/substance/833555#section=3D-Conformer, accessed on 20 July 2021) (Bethesda, MD, USA).

### 2.2. Animal Treatment

The Ethics Committee approved all experimental procedures used in the animal work on the Institutional Animal Care and User Committee of Chuang Hwa University of Medical Technology, Tainan, Taiwan, protocol A104-25 approval date: 31 December 2015. Eight-week-old male wild-type C57BL/6 mice were purchased from Jackson Laboratory (Bar Harbor, ME, USA) and fed standard laboratory chow. To establish a murine model of cisplatin-induced AKI, mice were intraperitoneally (i.p.) injected with 30 mg/kg of cisplatin (Sigma-Aldrich, Cat.# PHR1624) in a total volume of 200 µL diluted in sterile phosphate-buffered saline (PBS) [25]. To examine the therapeutic effects of GSK-3β blockade, mice were i.p. pre-injected with 5 mg/kg PBS-diluted BIO [20] in a total volume of 200 μL for 0.5 h. PBS was used as the vehicle control.

### 2.3. Immunohistochemistry

The deparaffinized and rehydrated tissue sections were quenched with 3% H_2_O_2_ in methanol. Antibody diluent (DAKO Corporation, Carpinteria, CA, USA) was used to dilute primary antibodies, including phospho-GS (pGS Ser641), phospho-GSK-3β (pGSK-3β Tyr216), CD3, Gr-1, CD11b, and CD54. Tissue sections were then stained with diluted antibodies at 4 °C overnight. Samples were washed twice in PBS and stained with HRP-labeled secondary antibodies 1 h at room temperature. Sections were developed with 3-amino-9-ethylcarbazole (AEC)-based staining. Sections were washed with PBS and visualized under a microscope (IX71; Olympus, Tokyo, Japan) or a fluorescence microscope (BX51; Olympus). We used the appropriate standard RGB intensity range for each cell marker to count the numbers of cells stained positively for CD3, Gr-1, CD11b, and CD54.

### 2.4. Pathological Examination

Mice sera were collected from peripheral blood via retro-orbital bleeding under pentobarbital anesthesia (40 mg/kg i.p.). An automatic biochemical analyzer (7080; Hitachi Koki Co., Ltd., Tokyo, Japan) was utilized for detecting serum levels of BUN and creatinine. For histopathological analysis, mice kidney tissues were harvested under a lethal overdose of pentobarbital (200 mg/kg i.p.) treatment. The kidney tissues were fixed, dehydrated, embedded in paraffin, and sliced, following deparaffinized in xylene, rehydrated in decreasing ethanol concentrations, stained with H&E, and viewed by light microscopy. The pathological changes, including renal cell necrosis, apoptosis, and cytoplasmic vacuolization, were validated accordingly [20,26].

### 2.5. Cell Culture

Human renal proximal tubular epithelial HK-2 cells (ATCC Cat# CRL-2190), which Dr. Y. Y. Chiou, Department of Pediatrics, National Cheng Kung University Hospital, kindly provided, were routinely grown on plastic in Dulbecco’s modified Eagle’s medium (DMEM; Invitrogen Life Technologies, Rockville, MD, USA) and Minimum Essential Medium (MEM; Invitrogen Life Technologies), supplemented with L-glutamine, HEPES, antibiotics, and 10% heat-inactivated fetal bovine serum (FBS; Invitrogen Life Technologies), and cultivated in a humidified atmosphere containing 95% air and 5% CO_2_.

### 2.6. Cell Death Assay

We utilized a colorimetric LDH activity assay (Cytotoxicity Detection kit; Roche Diagnostics, Lewes, UK), as diagnosed by a microplate reader (SpectraMax 340PC; Molecular Devices, Inc., Sunnyvale, CA, USA), to evaluate cell damage according to the manufacturer’s instructions. To measure apoptotic cells, TUNEL (terminal deoxynucleotidyl transferase dUTP nick end labeling) staining was performed using a TUNEL assay kit (ApopTag Peroxidase In Situ Apoptosis Detection Kit; Sigma-Aldrich) according to the manufacturer’s instructions. Furthermore, we also used Annexin V Apoptosis Detection Kit (BioLegend) to identify apoptotic and necrotic cells. Fluorochrome-labeled Annexin V can specifically target and identify apoptotic cells by interacting with phosphatidylserine in a calcium-dependent manner. Additionally, a counterstaining dye propidium iodide (Sigma-Aldrich) was used to distinguish between the necrotic and apoptotic cells as measured by fluorescent microscopy (BX51) and flow cytometry (FACSCalibur; BD Biosciences, San Jose, CA, USA) and FlowJo software (TreeStar, Ashland, OR, USA). Hoechst 33258 (Sigma-Aldrich) was used as a nuclear stain.

### 2.7. Western Blotting

Western blotting was carried out to measure protein expression according to the online support of Immunoblotting Protocol (Merck, Darmstadt, Germany) (https://www.sigmaaldrich.com/TW/en/applications/protein-biology/western-blotting, accessed on 20 July 2021). The proteins were subjected to standard SDS-PAGE and then transferred to a PVDF membrane (Millipore, Billerica, MA, USA). The protein membrane was blocked with 5% skim milk and then stained with diluted primary antibodies at 4 °C overnight. After being washed twice in PBS-Tween 20, the membrane was stained with diluted horseradish peroxidase (HRP)-conjugated secondary antibodies 1 h at room temperature. The blots were developed using the enhanced chemiluminescence solution (PerkinElmer Life Sciences Inc., Boston, MA, USA) according to the manufacturer’s instructions.

### 2.8. RNA Interference

A lentivirus-based shRNA was performed to silence the expression of human GSK-3β (TRCN0000040001: targeting sequence 5′-GCTGAGCTGTTACTAGGACAA-3′). In addition, targeting luciferase (shLuc) was used as a negative control of shRNA. All shRNA clones obtained from the National RNAi Core Facility, Institute of Molecular Biology/Genomic Research Center, Academia Sinica, Taipei, Taiwan, were prepared for cell transfection. Following 24 h post-transfection, cells were selected in a culture medium containing puromycin (Calbiochem, San Diego, CA, USA) for 3 days.

### 2.9. ELISA

According to the manufacturer’s instructions, the concentrations of cytokines/chemokines in the murine sera were determined using ELISA kits (R&D Systems, Minneapolis, MN, USA).

### 2.10. Statistical Analysis

The values are given as the mean ± standard deviation (SD). The groups were compared using Student’s two-tailed unpaired *t* test; significance was set at *p <* 0.05.

## 3. Results

### 3.1. Cisplatin Treatment Causes GSK-3β Activation Accompanied by Nephrotoxicity in an Experimental Murine Model of AKI

**Figure 1:**
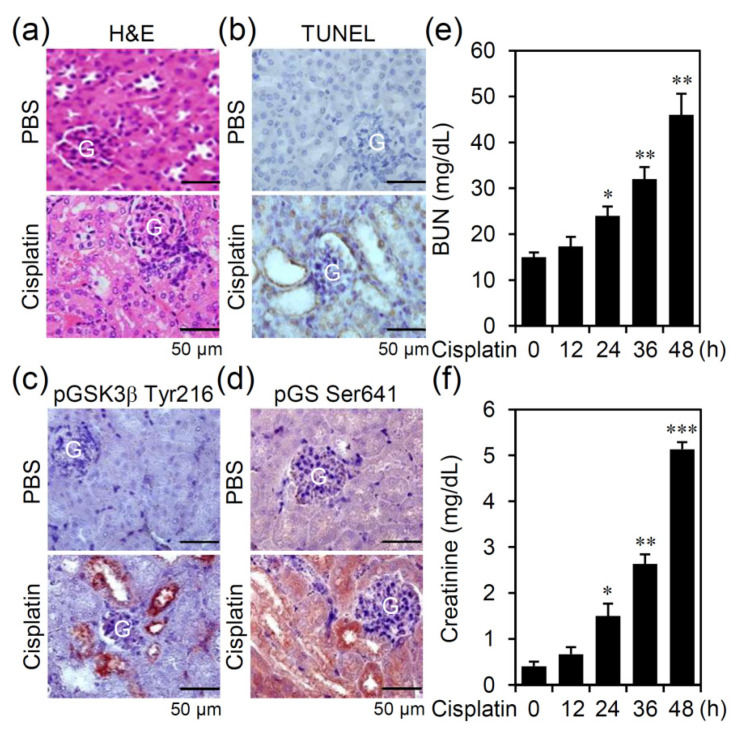
Cisplatin treatment causes GSK-3β activation in vivo accompanied by nephrotoxicity and tubular cell apoptosis. Male C57BL/6 mice (*n* = 3) were injected *i.p.* with cisplatin (30 mg/kg) for 24 h. (**a**) H&E staining showing histopathological changes. (**b**) TUNEL assay of apoptotic cells (*brown*). Hematoxylin plus AEC-based immunohistochemical staining showed the expression of phospho-GSK-3β (pGSK-3β) Tyr216 (**c**, *red*) and phospho-GS (pGS) Ser641 (**d**, *red*). For all of the images (scale bar = 50 μm), one representative set of data obtained from three mice is shown. G, glomeruli. Serum levels of (**e**) BUN and (**f**) creatinine were determined after cisplatin stimulation in a time-dependent manner. * *p* < 0.05, ** *p* < 0.01, and *** *p* < 0.001 compared with untreated cells.

To validate the pathological roles of GSK-3β for developing a different therapeutic strategy against AKI, we used the murine model of cisplatin-induced AKI in which mice received a single high dose of cisplatin (30 mg/kg). Drug treatment caused severe changes in histopathology on proximal tubular injury, including vacuolization, necrosis, immune cell infiltration (Figure 1a), and the induction of cell apoptosis (Figure 1b). Among the renal lesions, immunohistochemical staining showed that cisplatin induced GSK-3β activation, as demonstrated by protein phosphorylation at its active residue tyrosine 216 (Figure 1c) and accompanied by the presence of GS phosphorylation at serine 641 (Figure 1d), a substrate of active GSK-3β [18]. In peripheral blood, biochemical assays showed that cisplatin induced a significant (*p* < 0.05) increase in serum BUN (Figure 1e) and creatinine (Figure 1f) at 24 h post-stimulation followed by a time-dependent increased response. The results illustrate that cisplatin treatment causes GSK-3β activation in vivo, renal inflammation, nephrotoxicity, and renal dysfunction in cisplatin-induced AKI.

### 3.2. Pharmacologically Inhibiting GSK-3 Activation Ameliorates Nephrotoxicity and Renal Dysfunction in an Experimental Murine Model of Cisplatin-Induced AKI

**Figure 2:**
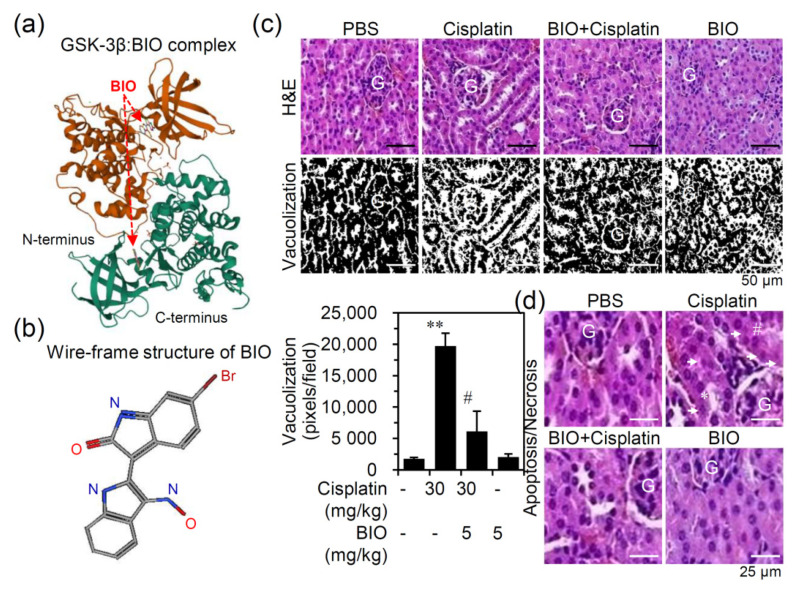
Inhibiting GSK-3 suppresses cisplatin-induced renal cytotoxicity. (**a**) The secondary structure of the GSK-3β:BIO dimer complex was adapted from the RCSB PDB 1UV5. (**b**) The wire-frame structure of BIO was adapted from PubChem 833555. (**c**) Male C57BL/6 mice (*n* = 3) were preinjected *i.p.* with the GSK-3 inhibitor BIO (5 mg/kg) for 0.5 h followed by cisplatin (30 mg/kg) for 24 h. Renal pathology was characterized based on H&E staining (scale bar = 50 μm) followed by ImageJ analysis to quantify vacuolization. ** *p* < 0.01 compared with untreated. # *p* < 0.05 compared with cisplatin. (**d**) Histopathological analysis of nephrotoxicity and cellular injury, including apoptosis (arrows), necrosis (#), and vacuolization (*). For all images, a representative image set obtained from three mice is shown (scale bar = 25 μm). G, glomeruli.

In this study, a synthetic ATP-competitive cell-permeable derivative BIO was used. The RCSB PDB 1UV5 and PubChem SID 833555, respectively, showed the secondary structure of the GSK-3β:BIO complex (Figure 2a) and the wire-frame structure of BIO (Figure 2b). GSK-3β comprises two lobes, including small N-terminal β sheets and large C-terminal α-helices. The ATP-binding pocket, composed of Arg96, Arg180, and Lys205, is located between the two lobes. The GSK-3β inhibitor BIO interacts within the ATP binding pocket to reduce its primed substrate phosphorylation on a GSK-3β-specific site. Following 0.5 h pretreatment with BIO (5 mg/kg) in established cisplatin-induced AKI, the histopathological analysis showed that cisplatin-induced renal tubular vacuolization (Figure 2c) and nephrotoxicity (Figure 2d), as shown by the presence of apoptotic/necrotic cells and cytoplasmic vacuolization [20,26], were significantly (*p* < 0.05) attenuated by treatment with the GSK-3 inhibitor BIO.

**Figure 3:**
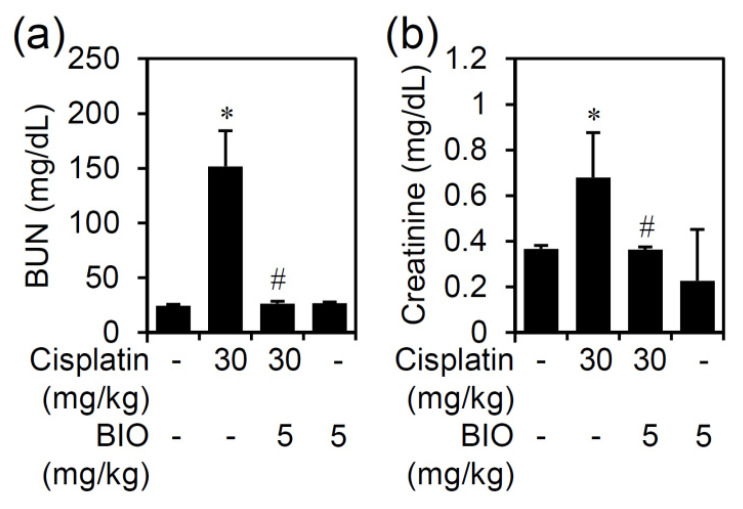
Inhibiting GSK-3 ameliorates cisplatin-induced AKI. Male C57BL/6 mice (*n* = 3) were preinjected *i.p.* with the GSK-3 inhibitor BIO (5 mg/kg) for 0.5 h followed by cisplatin (30 mg/kg) for 24 h. Serum (**a**) BUN and (**b**) creatinine levels were determined. Quantified data are the mean ± SD. * *p* < 0.05 compared with untreated. # *p* < 0.05 compared with cisplatin.

In cisplatin-induced AKI, we measured the increase in serum BUN and creatinine as the serological markers of renal injury. Following cisplatin treatment, the significantly increased BUN and creatinine could be detected accompanied by cisplatin-induced AKI. Furthermore, BIO treatment significantly (*p* < 0.05) inhibited the cisplatin-induced increase in serum BUN (Figure 3a) and creatinine (Figure 3b). Thus, the data demonstrate that pharmacological inhibition of GSK-3β effectively reduces cisplatin-induced nephrotoxicity and renal dysfunction in cisplatin-induced AKI.

### 3.3. Pharmacologically Inhibiting GSK-3 Activation Ameliorates Renal Inflammation in an Experimental Murine Model of Cisplatin-Induced AKI

**Figure 4:**
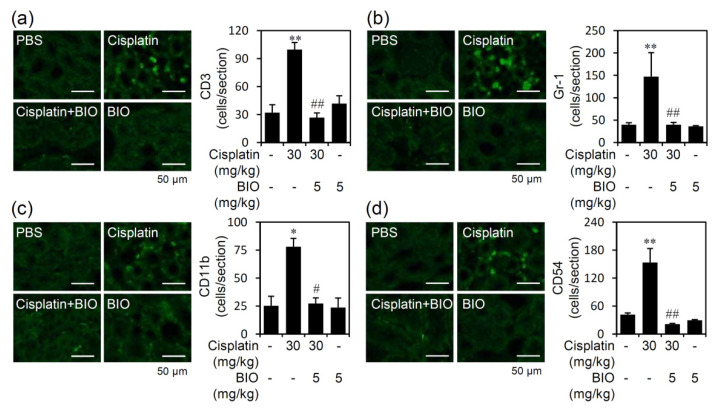
Inhibiting GSK-3 reduces cisplatin-induced renal immune cell infiltration. Male C57BL/6 mice (*n* = 3) were preinjected *i.p.* with the GSK-3 inhibitor BIO (5 mg/kg) for 0.5 h followed by cisplatin (30 mg/kg) for 24 h. Immunohistochemical staining for (**a**) CD3, (**b**) Gr-1, (**c**) CD11b, and (**d**) CD54 (as shown by the number of positive cells per section). For all images, a representative image set obtained from three mice is shown (scale bar = 50 μm). Quantified data are the mean ± SD. * *p <* 0.05 and ** *p <* 0.01 compared with untreated. # *p <* 0.05 and ## *p <* 0.01 compared with cisplatin.

We next studied the involvement of infiltrated inflammatory immune cells, such as neutrophils, monocytes/macrophages, T lymphocytes, and adhesion molecule CD54, which all are critical mediators of experimental cisplatin nephrotoxicity, in the animal model of cisplatin-induced AKI. In renal tissue slices of cisplatin-induced AKI, immunofluorescent staining showed a significant (*p* < 0.05) increase in the infiltration of CD3-positive T cells (Figure 4a), Gr-1-positive granulocytes (Figure 4b), and CD11b-positive monocytes/macrophages (Figure 4c). Additionally, cisplatin treatment significantly (*p* < 0.05) caused upregulation of CD54 (Figure 4d) in the kidneys. Notably, pretreatment with BIO significantly (*p* < 0.05) reduced cisplatin-induced immune cell infiltration and CD54 expression. Thus, in addition to renal cell protection, pharmacologically inhibiting GSK-3 effectively diminishes cisplatin-induced immune cell infiltration in cisplatin-induced AKI. 

**Figure 5:**
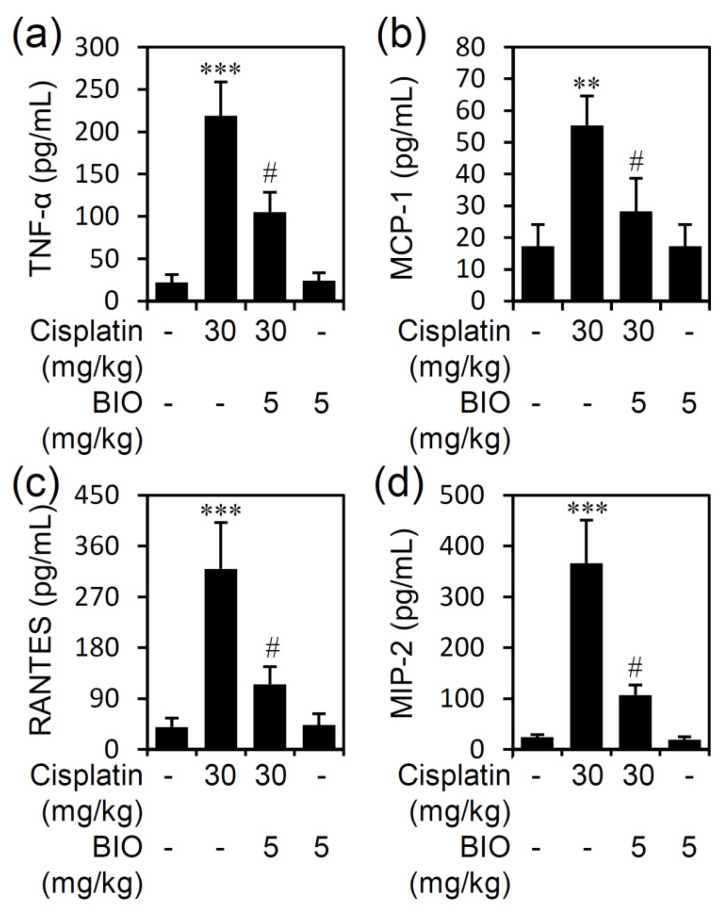
Inhibiting GSK-3 blocks cisplatin-induced proinflammatory cytokine/chemokine production. Male C57BL/6 mice (*n* = 3) were preinjected *i.p.* with the GSK-3 inhibitor BIO (5 mg/kg) for 0.5 h followed by cisplatin (30 mg/kg) for 24 h. ELISA for (**a**) TNF-α, (**b**) MCP-1, (**c**) RANTES, and (**d**) MIP-2. Quantified data are the mean ± SD. ** *p <* 0.01 and *** *p <* 0.001 compared with untreated. # *p <* 0.05 compared with cisplatin.

To investigate the induction of cisplatin nephrotoxicity by producing and activating pro-inflammatory cytokines and chemokines, ELISA analysis demonstrated significantly (*p* < 0.05) decreased levels of serum TNF-α (Figure 5a), monocyte chemotactic protein-1 (MCP-1) (Figure 5b), regulated upon activation of normal T cell expressed and secreted (RANTES) (Figure 5c), and macrophage inflammatory protein-2 (MIP-2) (Figure 5d) in BIO-treated mice following cisplatin stimulation. Thus, in addition to renal cell protection, pharmacologically inhibiting GSK-3 effectively diminishes cisplatin-induced renal inflammation in cisplatin-induced AKI.

### 3.4. Pharmacologically Inhibiting GSK-3 Activation Promotes Cell Survival from Cisplatin-Induced Renal Cell Cytotoxicity In Vitro

**Figure 6:**
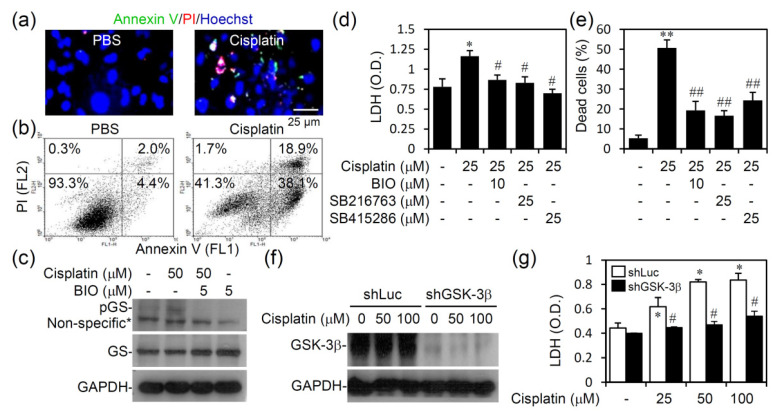
Cisplatin treatment causes GSK-3β activation in vitro followed by cell death. HK-2 cells were treated with cisplatin for 24 h in the absence or presence of a 0.5 h pretreatment with the GSK-3 inhibitor BIO. (**a**) Fluorescent image of Annexin V/PI/Hoechst 33258 staining for detecting apoptotic or necrotic cells. (**b**) Flow cytometric analysis of Annexin V/PI staining for detecting apoptotic or necrotic cells. (**c**) Representative Western blotting showed the expression of phospho-GS Ser641 and GS. GAPDH was used as an internal control. Cisplatin-stimulated HK-2 cells with or without pretreatment with the GSK-3 inhibitors BIO, SB216763, and SB415286 were assessed for (**d**) cytotoxicity by determining LDH release and for (**e**) apoptotic or necrotic cells by Annexin V/PI staining. (**f**) GSK-3β expression in shLuc- or shGSK-3β-transfected HK-2 cells. (**g**) The LDH activity assay detected cytotoxicity. For Western blotting, a representative data set obtained from repeated experiments is shown. Data are the mean ± SD of triplicate cultures. O.D., optical density. * *p <* 0.05 and ** *p <* 0.01 compared with PBS. # *p <* 0.05 and ## *p <* 0.01 compared with cisplatin-treated and shGSK-3β-transfected cells.

Our work demonstrates the presence of active GSK-3β in proximal tubular cells of cisplatin-treated kidneys. To investigate the possible role of GSK-3, an in vitro assay was performed. Immunostaining with Annexin V/propidium iodide (PI)/Hoechst 33258 followed by fluorescence microscopic observation showed distinct types of cell death, including apoptosis and necrosis, in cisplatin-treated HK-2 human renal proximal tubular epithelial cells (Figure 6a). Annexin V plus PI staining followed by flow cytometric analysis confirmed the imaging results (Figure 6b) that cisplatin caused cell apoptosis and necrosis in HK-2 cells. Western blot analysis showed that cisplatin caused GSK-3 activation in HK-2 cells, as detected by the phosphorylation of its substrate protein GS at serine 641, whereas pharmacologically inhibiting GSK-3 with the inhibitor BIO reduced this phosphorylation (Figure 6c). Notably, treatment with the GSK-3 inhibitors BIO, SB216763, and SB415286 significantly (*p* < 0.05) reduced cisplatin-induced cytotoxicity as detected by LDH release (Figure 6d) and apoptosis/necrosis as determined by Annexin V plus PI staining (Figure 6e). Knocking down GSK-3β with lentiviral-based shRNA (Figure 6f) confirmed that cisplatin significantly (*p* < 0.05) induced cytotoxicity in a GSK-3β-regulated manner (Figure 6g). These findings reveal a cytotoxic role of GSK-3β in cisplatin-induced AKI by facilitating renal cell death.

## 4. Discussion

In this study, inhibiting GSK-3β prevented nephrotoxicity in vivo and in vitro and suppressed inflammatory responses and renal dysfunction in cisplatin-induced AKI. Consistent with our findings, pharmacologically and genetically targeting GSK-3β confers cytoprotection against renal injury in several different in vivo models of AKI [15,20,21,22]. These findings indicate the involvement of GSK-3β signaling pathways in controlling cellular activation of cisplatin stimulation, such as DNA damage, oxidative stress, MAPK activation, and cell responses to cisplatin treatment, including renal cell death and inflammation. Therefore, GSK-3β activation caused by cisplatin as well as cisplatin-induced cytotoxic and inflammatory factors is hypothesized. Although active GSK-3β can facilitate cytotoxicity and inflammation in cisplatin-induced AKI, renal cell GSK-3β is suggested to be therapeutically targeted for ameliorating AKI.

Our in vivo study showed substantially increased expression of active GSK-3β in proximal tubular epithelial cells around the glomerulus under cisplatin treatment. Further in vitro studies confirmed that cisplatin directly activated GSK-3β in proximal tubular epithelial cells. All of the data showed that cisplatin treatment activates GSK-3β in vivo and in vitro; however, the possible molecular mechanisms of GSK-3β activation initiated by upstream factors and regulated by downstream signaling pathways remain unclear. Although inactivation of GSK-3β by PI3K/Akt signaling generally causes cisplatin resistance [27,28], it is hypothesized that cisplatin may deactivate PI3K/Akt to activate GSK-3β. Indeed, reversing cisplatin resistance by interfering with PI3K/Akt has been demonstrated in several studies [29,30]. In cisplatin-induced AKI, although we first demonstrated the activation of GSK-3β caused by cisplatin in vivo in kidneys and in vitro in proximal tubular epithelial cells, the potential regulation underlying cisplatin-induced GSK-3β activation in renal tubular cells and their effects on GSK-3β-modulated pro-apoptotic factors [14,31] as well as pro-inflammatory factors [17,18] requires further investigation.

In general, cisplatin-based chemotherapy causes nephrotoxicity directly by triggering tubular cell death and indirectly by exacerbating renal inflammation [32,33]. Despite aggressive hydration with PBS, renal impairment occurs during cisplatin-based chemotherapy. For the treatment of cisplatin-induced AKI [33], regulation on cisplatin-induced cell death by targeting Bax [34] and p53 activation [35], cell senescence and growth arrest by targeting JNK [36] and p38 MAPK [37], endoplasmic reticulum stress by targeting PERK and CHOP [38], autophagy by targeting mTOR [39], and immune cell activation by targeting CD4 positive T cells [40] and TNF-α [41] are proposed. Furthermore, therapeutic agents targeting cisplatin-induced DNA damage, ROS generation, and EGFR-Src-MAPK signaling are used to prevent renal cell death in cases of cisplatin-induced AKI [10,42].

We proposed that GSK-3β signaling pathways may mediate cellular activation of cisplatin stimulation, such as DNA damage, oxidative stress, MAPK activation, and cell responses to cisplatin treatment, including renal cell death and inflammation. Consistent with other studies showing that GSK-3 is involved in several nephrotoxicity models, including endotoxemia, ischemia, and non-cisplatin drugs [15,19,20,22], this study also provides evidence of cisplatin-induced GSK-3β activation in vivo and in vitro. Preventing renal cell death from cisplatin-induced cytotoxicity is therefore hypothesized to be effective against AKI. However, the greatest challenge for preventing cisplatin-induced AKI is that the marginal effects of inducing cell death are also the primary pharmacological basis of cisplatin as an anticancer agent. An exciting approach of inhibiting PKC-δ considerably reduces cisplatin-induced AKI without blocking its chemotherapeutic efficacy in mouse models of cancer [43,44]. This approach explicitly blocks cisplatin cytotoxicity in renal tubular cells but not in cancer cells. Accordingly, drugs used for all antiapoptotic approaches against AKI are needed to validate its effectiveness using a cancer model of cisplatin treatment.

Cisplatin-induced renal inflammatory responses, such as immune cell infiltration and cytokine/chemokine production, may marginally exacerbate nephrotoxicity with the synergistic reaction of cisplatin [12,45]. Consistent with previous studies [40,46,47,48], our study of histopathological examinations also identified increased infiltrated inflammatory immune cells, including neutrophils (Gr-1 positive), monocytes/macrophages (CD11b positive), and T lymphocytes (CD3 positive), increased CD54 expression, and overproduction of TNF-α and chemokines positively associated with cisplatin-induced AKI. GSK-3β also acts as a pro-inflammatory regulator by controlling several kinases, phosphatases, and transcription factors in inflammation [16,17,18]. For example, GSK-3β can mediate pro-inflammatory cytokine production by modulating NF-κB activation. Therefore, it is attractive to explore the possible mechanisms of GSK-3β-regulated cisplatin-induced renal inflammation and inflammation-associated nephrotoxicity. The cytopathogenic roles of pro-inflammatory cytokine TNF-α in facilitating renal cell inflammatory activation and cell apoptosis in cisplatin-induced AKI have been widely demonstrated [41,46,47,48,49]. Regarding the involvement of GSK-3β in controlling TNF-α expression [17,50] as well as TNF-α receptor signaling [14,51], it is hypothesized that GSK-3β inhibition ameliorates cisplatin-induced AKI through a mechanism involving the blockade of the TNF-α response.

A current study also reported BIO treatment, used as Wnt/β-catenin agonist, as a therapeutic strategy against cisplatin-induced AKI [52]. As consistent with our findings, cisplatin-induced nephrotoxicity and tubular cell apoptosis were inhibited by BIO treatment. In addition, the study also showed the potential blockade of BIO on cisplatin-induced ROS generation. Based on the findings, the increased Wnt/β-catenin signaling may facilitate cell survival against cisplatin cytotoxicity. As further demonstrated in our study, cisplatin effectively causes GSK-3β activation, mainly in renal tubular cells, accompanied by the induction of cytotoxicity as well as renal inflammation. On the other hand, the blockade of GSK-3β by using several GSK-3β inhibitors and the genetic shRNA approach shows renal cell protection and anti-inflammation from cisplatin stimulation. The pathogenic role of GSK-3β and its possible downstream responsive factors, not only Wnt/β-catenin but also Bax and p53 [14], related to cell survival and death, are therefore explored in cisplatin-induced AKI by our and previous research [19,34,35,52].

In conclusion, this study demonstrated GSK-3β activation caused by cisplatin in vivo in kidneys and in vitro in proximal tubular epithelial cells. Accompanied by active GSK-3β in cisplatin-induced AKI, increased inflammatory immune cell infiltration and cytokine/chemokine production were identified with the induction of renal cell cytotoxicity all highly associated with renal injury. Pharmacological inhibition of GSK-3 effectively ameliorated renal inflammation and nephrotoxicity in vivo in cisplatin-induced AKI. For the basis of anti-AKI, inactivation of GSK-3β rescued renal cell survival from cisplatin-induced renal cell cytotoxicity.

## Data Availability

The authors confirm that their article contains a data availability statement.
